# Biological Control Outcomes Using the Generalist Aphid Predator *Aphidoletes aphidimyza* under Multi-Prey Conditions

**DOI:** 10.3390/insects7040075

**Published:** 2016-12-14

**Authors:** Sarah E. Jandricic, Stephen P. Wraight, Dave R. Gillespie, John P. Sanderson

**Affiliations:** 1 Ontario Ministry of Agriculture, Food and Rural Affairs, Vineland Station, ON L0R 2E0, Canada; 2 Robert W. Holly Center for Agriculture and Health, Agricultural Research Service, USDA, Ithaca, NY 14853, USA; spw4@cornell.edu; 3 Pacific Agri-Food Research Centre, Agriculture and Agri-Food Canada, Agassiz, BC V0M 1A0, Canada; dave.gillespie@AGR.GC.CA; 4 Department of Entomology, Cornell University, Ithaca, NY 14850, USA; jps3@cornell.edu

**Keywords:** *Aulacorthum solani*, *Myzus persicae*, biological control in greenhouses, predator prey interactions, plant strata, prey preference

## Abstract

The aphidophagous midge *Aphidoletes aphidimyza* (Diptera: Cecidomyiidae) is used in biological control programs against aphids in many crops. Short-term trials with this natural enemy demonstrated that that females prefer to oviposit among aphids colonizing the new growth of plants, leading to differential attack rates for aphid species that differ in their within-plant distributions. Thus, we hypothesized that biological control efficacy could be compromised when more than one aphid species is present. We further hypothesized that control outcomes may be different at different crop stages if aphid species shift their preferred feeding locations. Here, we used greenhouse trials to determine biological control outcomes using *A. aphidimyza* under multi-prey conditions and at different crop stages. At all plant stages, aphid species had a significant effect on the number of predator eggs laid. More eggs were found on *M. persicae* versus *A. solani*-infested plants, since *M. persicae* consistently colonized plant meristems across plant growth stages. This translated to higher numbers of predatory larvae on *M. periscae*-infested plants in two out of our three experiments, and more consistent control of this pest (78%–95% control across all stages of plant growth). In contrast, control of *A. solani* was inconsistent in the presence of *M. persicae*, with 36%–80% control achieved. An additional experiment demonstrated control of *A. solani* by *A. aphidimyza* was significantly greater in the absence of *M. persicae* than in its presence. Our study illustrates that suitability of a natural enemy for pest control may change over a crop cycle as the position of prey on the plant changes, and that prey preference based on within-plant prey location can negatively influence biological control programs in systems with pest complexes. Careful monitoring of the less-preferred pest and its relative position on the plant is suggested.

## 1. Introduction

Although multiple prey species can potentially have positive outcomes for biological control programs using natural enemies [1] the reverse can also be true (e.g., [2]). Negative outcomes can occur through preferential attack of predators on one herbivore in a food web, deflecting predation away from other pests [3]. Interestingly, this phenomenon can be mediated by preferred feeding locations of different prey species, rather than an inherent preference of the natural enemy for one prey type over another. For example, the presence of the bird-cherry oat aphid, *Rhopalosiphum padi*, decreases the efficacy of lacewings for controlling the Russian wheat aphid, *Diuraphids noxia*, as a direct result of *R. padi* feeding in more predator-accessible locations on the plant [4,5]. Within the time-scale of a crop cycle, such unbalanced predation due to within-plant distribution differences of aphid species could lead to apparent mutualism. Specifically, repeated attacks on the preferred pest lower the fitness of the first pest species, while simultaneously resulting in reduced control (and increased fitness) of the second [3,4], leading to differential species control. In systems where multi-species pest outbreaks are common (e.g., greenhouse crops), the potential for unbalanced pest control should be investigated carefully for specific natural enemies before commercial suppliers recommend a product for an entire group of pests. Otherwise, failure of biological control for one or more species may occur. Concerns about failure of biocontrol programs is cited as one reason growers hesitate to adopt this pest control technique [6].

*Aphidoletes aphidimyza* (Rondani) (Diptera: Cecidomyiidae) is a commercially available natural enemy of aphids (Hemiptera: Aphididae) that is released in a surprisingly broad array of crops. These include certain field crops (e.g., alfalfa, hops), orchards (e.g., apples), as well as in greenhouse and nursery operations in North America and Europe [7]. In greenhouses, it is mostly released in crops such as pepper and tomato, potted ornamentals, and woody ornamentals [7]. Despite the low threshold for insect presence/damage in ornamentals, *A. aphidimyza* does have the ability to be an effective biocontrol agent in this system [8]. It is sold as a “generalist” aphid predator, and is reported to feed on over 75 different aphid species [9].

However, a collection of previous research demonstrates that *A. aphidimyza* oviposits preferentially in aphid colonies that occupy new growth of plants (especially meristematic tissue) compared to other plant locations [10,11]; and recently [12]. Specifically, Jandricic et al. 2013 [12] demonstrated that the foxglove aphid (*Aulacorthum solani* (Kaltenbach)), using lower leaves of vegetative plants as its primary feeding site, received fewer *A. aphidimyza* eggs than plants infested with green peach aphid (*M. persicae* (Sulzer)), which aggregated on plant meristems. These results suggest that *M. persicae* may have the ability to interfere with control of *A. solani* when present in the same crop; the same may also be true of other aphid species combinations. Currently, however, the ability of *A. aphidimyza* to control simultaneous outbreaks of these pests remains poorly understood. Extrapolations from oviposition-preference studies of predators alone cannot be relied upon, since factors such as prey suitability (e.g., [13]) prey size (e.g., [14,15]), prey intrinsic rate of increase, larval competition in predators [16,17], prey defensive/predator avoidance behaviors [15], prey toxin sequestration [18], age of aphid colonies (e.g., [19,20]), and others factors could all potentially impact predation rates once eggs hatch [15].

Further complicating biological control in a program relying on a predator with prey preferences based on prey feeding locations is that within-plant prey distributions can change over time. Previous greenhouse studies show that aphids often move up the plant when the plant becomes reproductive [21,22,23]. A greater understanding of how a specific natural enemy responds to different pest distributions is needed if a biological control program is to be reliable over entire cropping cycles. This is especially needed situations where crops are of high value, where multi-species outbreaks occur frequently, and where crop cycles are short—all descriptions of greenhouse floriculture crops.

Thus, the objectives of this paper were to determine (1) how distributions of our model aphid species changed when infesting a greenhouse ornamental crop at different growth stages; (2) the response of *A. aphidimyza* to changes in aphid species distributions; (3) the ability of *A. aphidimyza* to curatively control multi-species aphid outbreaks across crop stages in an ornamental crop; and (4) how the presence of a more preferred prey species (*M. persicae*) impacts control outcomes of a second target pest (*A. solani*). Studies were of longer duration than Jandricic et al. 2013 [11] (here, we follow *Aphidoletes* from egg hatch to pupation) to evaluate whether oviposition preferences indeed translate to significant differences in control of two disparate aphid species. These results are then applied to practical aphid control in floriculture greenhouse crops.

## 2. Materials and Methods

### 2.1. Insects

Mixed clonal populations of both aphid species (*M. persicae* and *A. solani*) were collected in Ithaca, NY in 2009 and were continuously reared on pansy (*Viola × wittrockiana* Gams.), as in Jandricic et al. (2010) [24]. Adult aphids for all experiments were selected directly from colonies, and were therefore of unknown age. Pansies were chosen as they are readily accepted as hosts by both aphid species, and are a type of spring bedding crop that regularly sees aphid infestations in commercial operations.

*A. aphidimyza* pupae were obtained from Applied Bio-Nomics Ltd. (Victoria, BC, Canada) for all experiments. Upon receipt, pupae were placed in emergence cages as described in Jandricic et al. (2013) [12]. Adult midges were used instead of pupae to provide better management of the actual number of adult flies released. Adult midges were not used in experiments until ca. 60 h post emergence to ensure mating had occurred and that females reached maximum egg production potential [25]. For each experiment, adult midges were collected from emergence cages with a mouth aspirator using glass vials to prevent midges from being injured due to static electricity. A subsample of 50–100 individuals was also taken from the *A. aphidimyza* emergence cage at the time of each experiment to determine sex ratio. The average sex ratio over experiments in Section 2.4, Section 2.5 and Section 2.6 was 1 male: 1.8 females (range = 1:1.5 to 1:1.9). However, a lower ratio of females was seen in the experiment assessing *M. persicae* presence/absence on *A. solani*, with an average of 1 male: 0.9 females.

### 2.2. Plant Material and Growth Stages Tested

For all experiments, pansy (*Viola × wittrockiana* Gams, var. Majestic giant II; Stokes Seeds, Buffalo, NY, USA) was used as the aphid host plant. Plants were grown as in Jandricic et al. (2010) [24]. Pansies were generally free of any additional ornamental pests (e.g., thrips, spider mites) throughout the experiments, and thus were free of any additional chemical or biological control treatments that may interfere with experimental outcomes.

Pansy crop stages tested included vegetative, budding, and flowering (produced under natural day length). Plants were considered vegetative as long as new growth at the meristem was not producing buds at the time of *A. aphidimyza* oviposition. To be considered budding, plants had to have at least one distinct bud forming at the apical meristem with buds being developed enough to have distinct petal tissue. Fewer than 25% of plants had a completely open flower at the end of the experiment. To test the flowering stage, plants (potted for ca. 8 weeks) had at least one fully open flower and one other flower bud on a tall stalk close to opening; plants continued to flower over the course of the experiment.

### 2.3. Experimental Set-Up

#### 2.3.1. Effects of Plant Stage on Multi-Species Aphid Control by *A. aphidimyza*

See Table 1 for details on experimental set-up. To determine what effect plant growth stage has on aphid distributions and *A. aphidimyza* response, the following experiments were conducted in separated, identical greenhouse compartments (2.75 m × 7.30 m) at the USDA-ARS facility in Ithaca, NY, USA. Greenhouse benches (0.92 m × 2.44 m) were used as blocks. This experimental design was repeated across three stages of plant growth: vegetative, budding and flowering. Experiments at different stages of plant growth were conducted separately due to experimental design constraints. Experiments were conducted across 2–3 greenhouse compartments, each in either spring or fall to provide similar growing conditions. In all cases, environmental controls were set to 24 °C day time temperature and 18 °C night time temperature and conditions were monitored with HOBO data loggers (Onset Computer, Bourne, MA, USA).

There were two types of aphid infested plants within each compartment: *M. periscae* or *A. solani*-infested plants. These plants were then subjected to one of two treatments: no treatment (control) or exposure to the same population of *A. aphidimyza*. Aphid species were not combined on the same plants since this would complicate choice results of the predator, and because this rarely occurs in commercial greenhouses ornamental crops. (Specifically, multiple aphid species infestations can occur simultaneously within the same crop within the same greenhouse, but they hardly ever colonize the same plants [26]).

To produce aphid infested plants, separate plants received either three adult *M. persicae* or five adult *A. solani* per plant; these numbers were chosen to ensure that densities of each aphid species would be similar at the time of *A. aphidimyza* release. Based on previous experiments, starting densities would result in ca. 40–50 aphids per plant on day 1 of the experiment (a moderate infestation). Aphids were added to the center of plants by fine brush and allowed to naturally distribute and reproduce on plants in the greenhouse for 1 week prior to the start of experiments (day 0).

Aphid infested plants were then placed in one of two locations: either out on the bench (to be exposed to predators), or within 61 cm × 61 cm × 61 cm cages (i.e., the controls; one cage at the end of each bench; BugDorm 2, BioQuip Products, Rancho Dominguez, CA, USA). Infested plants intended for predator exposure (three/aphid species/bench) were placed in random positions in a configuration of four rows of seven plants (28 total), with ca. 15 cm spacing to prevent transfer of aphids. The remaining 22 plants were left un-infested (to serve as “background” plants to force *A. aphidimyza* to search for prey, as in a commercial greenhouse). Plants within cages (three/aphid species/bench) were also maximally spaced to prevent aphid transfer.

On day 0 of all experiments, 100 adult *A. aphidimyza* midges were released in each compartment just prior to dusk, as per commercial recommendations. Thus, the same *A. aphidimyza* population had a choice of *M. persicae* or *A. solani*-infested plants randomized on benches. Predators were released at the center point of each grid of plants on each bench, as in Jandricic et al. (2013) [12]. The total release rate used per compartment was 2.5× the high-release rate of two midges/m^2^ suggested by commercial biocontrol companies, which is equivalent to a predator: prey ratio of 1:10 at the time of release. This is an intermediate rate among those that have been found effective in previous testing in greenhouse crops (see [27]). Ventilation fans were turned off overnight to promote midge settling in the crop. To increase relative humidity (RH) to promote oviposition (see [28]), mist emitters, located beneath each bench, were operated for 5 min of every 60 min for the duration of the experiment. Aphid and predator numbers were then sampled across three separate dates in all four treatments (see Insect Sampling, below, Section 2.4).

#### 2.3.2. Effect of *M. persicae* Presence/Absence on Control of *A. solani* by *A. aphidimyza*

To determine if *A. solani* control by *A. aphidimyza* is improved in the absence of alternative prey, greenhouse compartments were set up with either (1) both *M. persicae* and *A. solani-*infested plants (two compartments); or (2) *A. solani*-infested plants only (two compartments). The number of infested plants was doubled within the *A. solani*-only compartment to present the predator with the same initial aphid densities in both treatments (i.e., 48 aphid-infested plants per compartment: 24 exposed to predators, and 24 serving as controls within cages). As individuals of *A. aphidimyza* from the same population (i.e., rearing batch) were released in the compartments, and all compartments were treated at the same time (and set to the same environmental conditions), we considered plant to be the experimental unit, not greenhouse (as with experiments above). All other experimental conditions/procedures were the same as above (Section 2.3.1).

### 2.4. Insect Sampling across All Experiments

For each experiment, one aphid infested plant/bench/treatment/species/compartment (see Table 1) was destructively sampled on each of three sample dates: day 2 after *A. aphidimyza* release, in order to assess density and location of aphids and the majority of *A. aphidimyza* eggs; day 6 after release, to assess aphid density and numbers of small *A. aphidimyza* larvae (given that eggs take ca. 3 days to hatch, larvae on this day would be ca. 2–3 days old) and day 9 after release, to make final assessments of aphid density on treatment and control plants at a point when the oldest of the *A. aphidimyza* larvae (5–6 days old at this point) had potentially begun to pupate, therefore reducing control potential.

On day 9, counts of large larvae still foraging on the plant were made, as were counts of any small larvae present from later oviposition. Though larval sizes were not distinguished during counts, the majority of larvae sampled on days 9–11 were large (e.g., based on our egg counts after the first 2 days of initial oviposition, small larvae could be estimated to be ≤20% of the larval population in all cases).

Along with numerical counts, aphids, predator eggs, and larvae were also recorded as being on one of several possible within-canopy “locations”. For vegetative plants, these locations consisted of bottom, middle, or top leaves or the center growing point of the plant. The respective leaves were ca. 0–2, 2–5, and >5 cm from the soil surface (with plants generally being 6–8 cm tall). The center growing point (henceforth referred to as the meristem) was specifically defined as the plant material remaining after all mature leaves were removed from the plant; it consisted of many small, immature leaves in a cluster comprising the central meristem and several small, under-developed lateral meristems (<2 cm in length). Aphids were rarely found on mature, vegetative stem tissue.

In the first compartment sampled with plants that were in the budding stage, aphids and *A. aphidimyza* on buds were included in counts of meristems as a type of “new growth”. However, for the following two compartments, insects on flower buds were tallied separately to characterize the attractiveness of this plant organ (this data is reflected in the distribution graphs in Section 3.1.2). For flowering plants, locations of flower buds, fully open flowers, and senescing flowers (i.e., visibly wilted and many having dropped petals) were included along with the locations described previously.

### 2.5. Environmental Conditions across Experiments

Average temperatures in the research greenhouse compartments from the point of *A. aphidimyza* release to the end of the experiment were similar between vegetative and flowering pansies; temperatures were somewhat higher for budding pansies. The average daily temperature ranged from 20 °C (vegetative and flowering plants; range: 12–26 °C) to 25 °C (budding plants; range: 16–35 °C). The average RH was 67%–86% across all experiments, being lowest in the experiment on flowering plants. In all cases, conditions in the cage were extremely close to ambient conditions in the greenhouse, and thus are not reported separately.

### 2.6. Statistical Analyses

To analyze the initial within-plant distributions of our aphid species at different plant growth stages (Section 3.2.1), a mixed model ANOVA was conducted on the proportion of total aphids found in each stratum on day 2 of the experiment. Plant stratum, aphid species and their interaction were tested. Proportions were used rather than numbers to facilitate comparisons between species even when at vastly different densities. Because proportions would sum to 100% for all strata, violating the assumption of independence, we omitted data from the medium stratum from all analyses. The medium stratum was selected for exclusion because it generally contained the lowest number of aphids. Proportional data were arcsine transformed to better meet the assumptions of the ANOVA. We also specified plant as the repeated measure from which location measurements were taken. The entire analysis was repeated with data subject to the empirical logit transformation, as recent simulations have indicated it may be an improvement over arcsine for proportions [29]. However, we detected no significant differences in outcomes between the two transformations; the results of the arcsine are reported.

Predator response (Section 3.1.2) was analyzed both between-plants and within-plants. For the between-plant analysis, numbers of eggs and larvae per plant were initially modeled against aphid species, as well as aphid density (since density is known to potentially affect *A. aphidimyza* oviposition, and initial aphid numbers were within a similar range, but not identical per plant), as well as their interaction. However, the interaction term was non-significant for each test, and density was only significant in one case (for eggs on vegetative plants, at *F*_1,20_ = 4.59, *p* = 0.045). Thus (with the exception of the case noted above) a reduced model was tested and only the effect of species is presented. In all cases, greenhouse compartment, and greenhouse bench (nested within compartment), were included as random effects to control for compartment to compartment (and block to block) differences. If data did not meet assumptions of the ANOVA, both aphids and eggs were log_10_(*x* + 1) transformed (hereafter referred to as a log–log transformation). Distribution of predator eggs within-plants was modelled as with aphids, above, on both day 2 and day 6.

To determine control outcomes (Section 3.2 and Section 3.3), we used data from the last day of each experiment. For each plant stage, effects of treatment, aphid species, and their interaction on the response of aphid numbers per plant was analyzed using a mixed-model ANOVA (PROC MIXED in SAS) [30]. Data were log_10_(*x* + 1) transformed prior to analysis, to better meet assumptions of the ANOVA. Random effects were the same as listed previously (with greenhouse compartment accounting for 0%–32% of the variability). In all cases, there was a significant interaction between treatment and species in the global analysis (*p* < 0.05). Thus, data were further investigated within both species and treatment using a Tukey–Kramer multiple means comparison. Our mixed-model ANOVAs approximately met assumptions of variance, but were not ideal. Thus, we also ran non-parametric *t*-tests to compare within-species and within-treatment effects. In all cases, non-parametric tests agreed with our Tukey–Kramer results, therefore the results of the parametric test are reported. All tests were done on log-transformed data, although untransformed means and standard errors are presented.

## 3. Results

### 3.1. Within-Plant Colonization of Insects at Different Plant Stages

#### 3.1.1. Prey Insects

**Figure 1:**
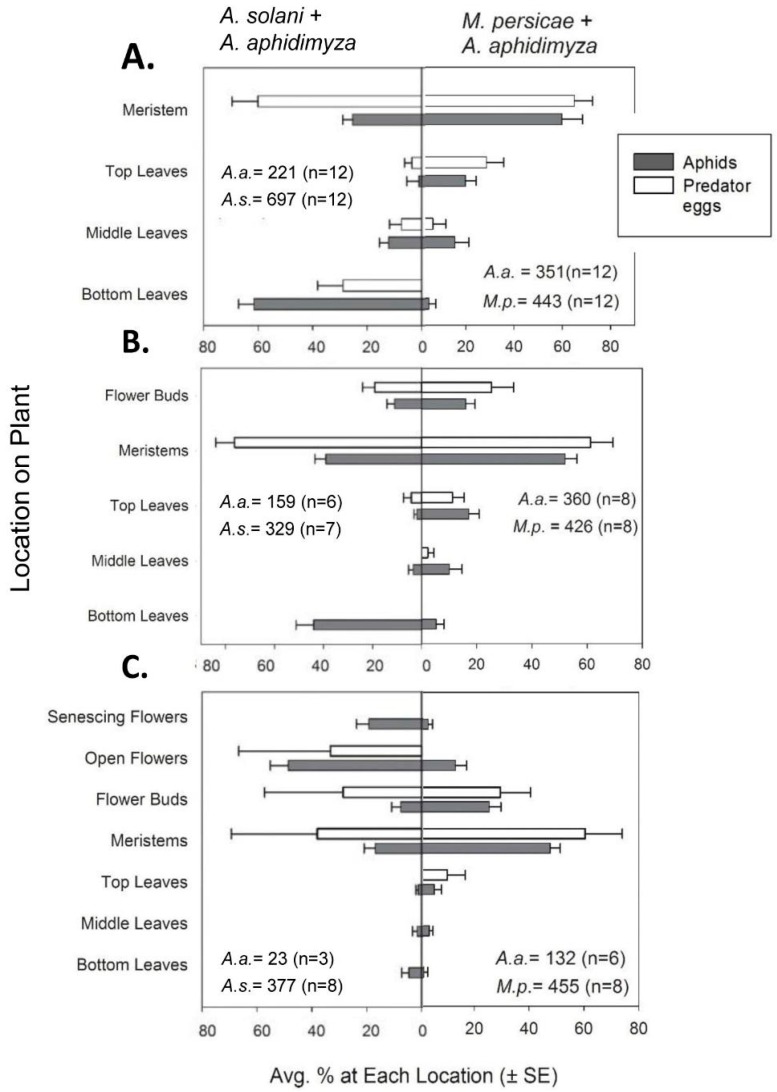
Plants were in the following stages of growth: (**A**) vegetative; (**B**) budding; and (**C**) flowering. Distribution (average % ± SE) of the total aphid population found at each location within plants are shown for *Aulacorthum solani* (*A.s*.) and *Myzus persicae* (*M.p*.) as black bars; proportion of total *A. aphidimyza* eggs deposited at each plant location is shown by the white bars. Total aphid and predator egg numbers for all samples are given. All results are from day 2 after release of 100 *A. aphidimyza adults*/compartment.

At all plant stages, within-plant distribution of our two aphid species differed on sampling day 2 (9 days after initial placement on plants; Figure 1). This is indicated by significant species-by-plant strata interactions for all tests (*F*_3,79_ ≥ 26.8, *p* ≤ 0001). *Myzus persicae* was found mainly on meristems at all plant stages (*t*_17.3_ ≥ 4.01 and *p* ≤ 0.0015 for all tests vs. meristems). Distributions of *A. solani* varied with plant growth stage (Figure 1).

On vegetative plants, most *A. solani* were found colonizing lower leaves (62%; *t*_63_ ≥ 4.93, *p* ≤ 0.002 for all comparisons; Figure 1A). However, on budding plants, populations were concentrated ca. equally on the meristems and bottom leaves (39% vs. 44% of the population, respectively; *t*_24_ = 0.41, *p* = 0.98 for this comparison; Figure 1B). On flowering plants, initial *A. solani* populations were concentrated almost entirely on flowers and buds (Figure 1C), with greatest proportions on open flowers (76% of the population *t*_23.2_ ≥ 3.37, *p* ≤ 0.0233 for all comparisons).

#### 3.1.2. Predators

**Figure 2:**
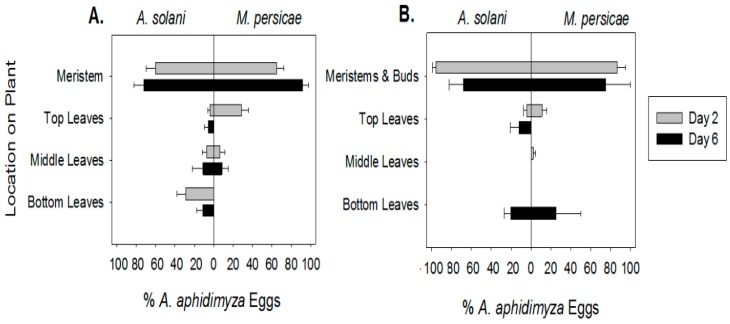
Within-plant distribution (average % ± SE) of *Aphidoletes aphidimyza* eggs on *Myzus persicae* or *Aulacorthum solani* 2 days and 6 days after release. (**A**) Oviposition on vegetative plants; (**B**) oviposition on budding plants.

Since >73% of all *A. aphidimyza* eggs were laid in the first 48 h in all experiments, the ANOVA was conducted on these data. In our within-plant analyses, plant strata were always significant for oviposition (*F*_2,41_ ≥ 27.8, *p* ≤ 0.0001). Species as a main effect was never significant (*F*_1,30_ = 1.24, *p* ≥ 0.27 in all cases). The interaction between species and strata was only significant in the case of vegetative plants (*F*_2,41_ = 8.08, *p* = 0.001). As in Jandricic et al. 2013 [12], higher number of *Aphidoletes aphidimyza* eggs were found within aphid colonies on meristems across all plant growth stages on day 2 (Figure 1; *t*_25.2_ ≥ 3.88, *p* ≤ 0.0046 for all tests). Additionally, for both aphid prey species, distribution of eggs deposited later in the experiment were similar to those deposited in the first 48 h (Figure 2). Most eggs were again oviposited on meristems despite the presence of conspecific eggs and larvae (*t*_11.6_ ≥ 3.32 and *p* ≤ 0.0116 for all comparisons with both aphid species on day 6).

**Figure 3:**
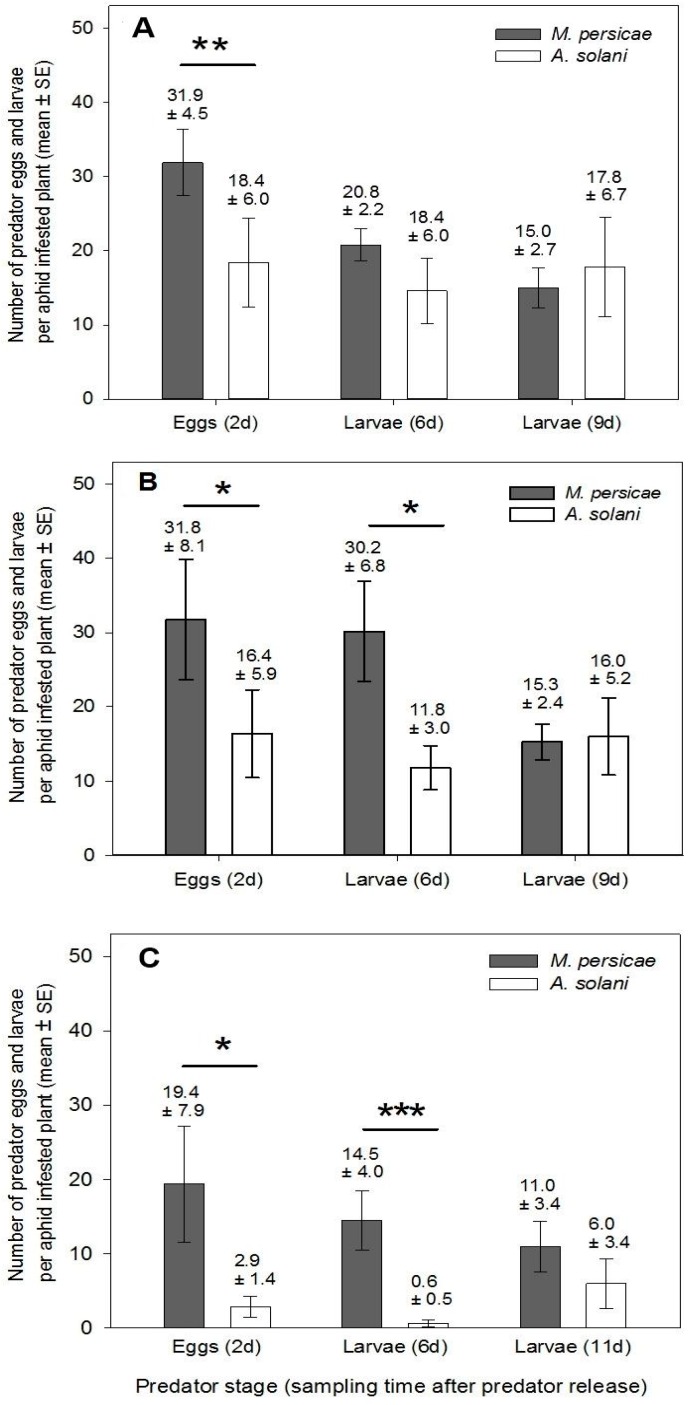
Mean number ± SE of *A. aphidimyza* eggs and larvae found on aphid infested plants 2d, 6d or 9–11 days after release of *A. aphidimyza* adults. Plants were (**A**) vegetative; (**B**) budding; or (**C**) flowering. Stars indicate significant differences between aphid species within sampling day. Any eggs deposited around day 6 likely did not contribute to overall control during the experiment time frame, thus data are not shown. Primarily large larvae were present on days 9–11 as the population was aging. A “*” symbol indicates a *p* ≤ 0.05, “**” indicates a *p* ≤ 0.01 and “***” indicates a *p* ≤ 0.001. Statistical tests were conducted on log-transformed data, though arithmetic means and SEs are shown.

Our between-plant tests demonstrated that higher numbers of eggs were consistently deposited on *M. persicae*-infested plants (Figure 3; *F*_1,14_ ≥ 4.71, *p* ≤ 0.047 for the effect of species on all plant stages), likely due to the higher number of aphids found on meristems in these plants. This also translated to higher numbers of young predator larvae on *M. persicae*-infested plants as the experiment went on for two out of our three plant stages (Figure 3: *F*_1,22_ ≥ 6.2, *p* ≤ 0.0206 on budding and flowering plants). However, by day 9, numbers of larvae were not statistically different between species (*F*_1,21_ ≤ 1.99 and *p* ≥ 0.1731 across all plant stages). This may be a result of larger larvae pupating earlier in the *M. persicae* treatment, since most of the aphids had already been eaten. Specifically, nine of 12 plants in both the vegetative and budding stages had ≤8 *M. persicae*/plant left on day 9; for *A. solani*, only 2–4 plants/growth stage had such low numbers.

### 3.2. Control Outcomes

#### 3.2.1. Vegetative Plants

**Figure 4:**
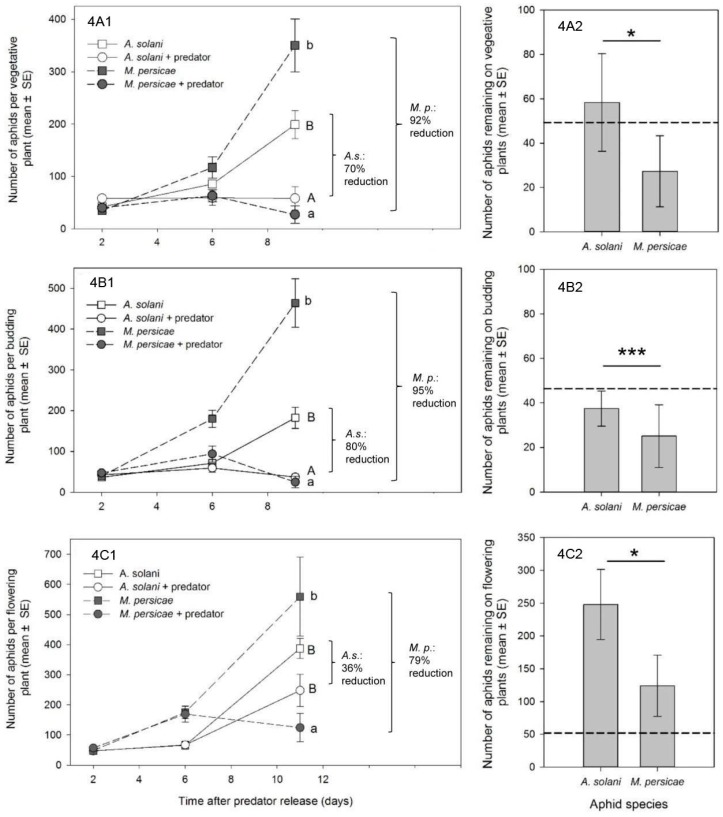
Plants were in the following stages of growth: (**A**) vegetative; (**B**) budding; (**C**) flowering. (**A1,B1,C1**) Mean aphid density ± SE of *A. solani* (*A.s.*) or *M. persicae*-infested (*M.p*.) plants with no control measure (square symbols) or with releases of *Aphidoletes aphidimyza* (circle symbols). Different letters represent significant differences from comparisons made within each aphid species. Percent control of each aphid species is also presented; (**A2,B2,C2**) Comparison between aphid species on predator-treated plants at the end of the experiments (means and SE presented). The dashed line represents the level of aphid infestation at the start of the experiment. A “*” symbol indicates weak significance (*p* ≤ 0.08); “***” indicates high significance (*p* ≤ 0.001). Statistical tests were conducted on log-transformed data, though arithmetic means and SEs are shown.

Treatment with *A. aphidimyza* had a significant effect on aphid numbers, but there was a significant interaction with species (Table 2). Applications of *A. aphidimya* resulted in reduced *M. persicae* relative to their controls (Figure 4A1; *t*_41.2_ = 7.45, *p* < 0.0001). Overall, 92% control of this species was achieved (27 ± 16.8 vs. 350 ± 50.6 aphids/plant). However, the predator was less effective against *A. solani*; percent control was significant (*t*_41_ = 4.15, *p* = 0.0009) but reached a maximum of just 70% (58 ± 22.7 vs. 199 ± 26.6 aphids/plant). Though numbers of *A. solani* and *M. persicae* remaining on plants at the end of the experiment were not significantly different (LSmeans = 1.36 ± 0.23 vs. 0.80 ± 0.23; *t*_41_ = 2.49, *p* = 0.077), only the population of *M. persicae* was reduced to below initial infestation levels.

#### 3.2.2. Budding Plants

As with vegetative plants, both predator treatment and treatment × species were significant (Table 2). On day 9, average numbers of *M. persicae*/plant in the *A. aphidimyza* treatment were reduced to almost half of initial densities (Figure 4B1), with numbers on seven of 12 plants reduced to ≤2 aphids/plant. Control of *M. persicae* was significant (25 ± 14.2 *M. persicae*/plant in the predator treatment vs. 464 ± 59.5 in the controls; *t*_41.5_ = 10.79, *p* ≤ 0.001) and represented the best control outcome across all experiments (95%).

Average *A. solani* per plant in the *A. aphidimyza* treatment was also significantly lower than the control (37 ± 7.9 vs. 182 ± 25.9 aphids/plant; *t*_41.4_ = 4.41, *p* = 0.0004). This represented 80% control, and, similar to *M. persicae*, was the best control outcome seen for *A. solani* across all experiments.

For both species, this was the only plant growth stage where pest abundance was reduced below initial starting levels. However, again, levels of *M. persicae* were still ultimately lower than *A. solani* (Figure 4B2; *t*_41.4_ =4.36, *p* = 0.0006; LSmeans = 1.50 ± 0.24 vs. 0.76 ± 0.24).

#### 3.2.3. Flowering Plants

Treatment and treatment × species were once again significant (Table 2). Consistent with the other plant stages, a higher percentage control was observed for *M. persicae* vs. *A. solani*: 78% for *M. persicae* (significant at: *t*_25.4_ = 3.90, *p* = 0.0033) vs. just 36% for *A. solani* (control was non-significant at: *t*_25_ = 1.33, *p* = 0.55). Here, neither aphid species was reduced below initial levels (Figure 4C1). However, final numbers of *M. persicae* in the predator treatment were once again lower than *A. solani*, though this was only weakly significant this time in both the parametric and non-parametric tests (Figure 4C2; LSmeans = 2.30 ± 0.15 vs. 1.78 ± 0.15; *t*_25_ =2.45, *p* = 0.08; Z = −1.73, *p* = 0.08).

### 3.3. Effect of M. persicae Presence/Absence on Control of A. solani by A. aphidimyza

**Figure 5:**
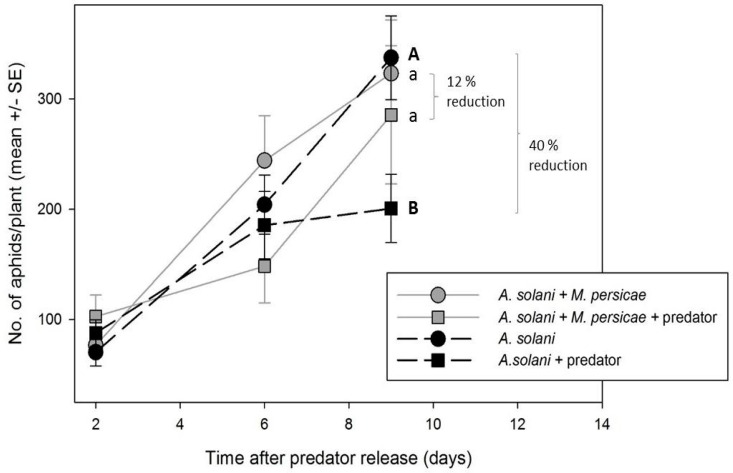
Mean number of *A. solani* per plant over time in the presence (square symbols) or absence (round symbols) of the predator *Aphidolotes aphidimyza* when presented alone or with a second, more preferred aphid species. Different letters indicate significant differences between treatments when *M. persicae*-infested plants are either present (**grey** symbols) or absent (**black** symbols). Statistical tests were conducted on log-transformed data, though arithmetic means and SEs are show.

In compartments with *M. persicae*, the predator had no significant impact on numbers of *A. solani*/per plant (*F*_1,19.3_ = 0.85, *p* = 0.4073), with only a 12% reduction (Figure 4C). However, when *A. solani* was the only aphid species in the greenhouse (presented at similar densities to the mixed aphid treatments), there was a significant predator effect (*F*_1,22.6_ =9.54, *p* = 0.0053), resulting in 40% control with *A. aphidimyza* present (Figure 5).

## 4. Discussion

This study demonstrated that, even when using a polyphagous aphid predator, differential control between greenhouse aphid species is possible. Building on the study by Jandricic et al. 2013 [12], this appears to be the result of a combination of within-plant oviposition preferences by the predator, coupled with varying within-plant distributions between aphid species that held true over all stages of plant growth. These results provide practical information for the use of *A. aphidimyza* for multi-species aphid outbreaks in greenhouse crops, a common problem for growers, but one receiving little study.

In our studies, new growth was the most prevalent canopy feeding location for *M. persicae* at all crop stages, and we saw a consistently high percentage control of *M. persicae* by *A. aphidimyza*—near or >80%—in all cases. In contrast, control of *A. solani* with this predator varied by plant growth stage. Knowing that *A. aphidimyza* prefers to attack aphid colonies on meristems [11,12], shifts in aphid within-plant locations across growth stages appears to be the main cause of variable control of *A. solani*. The highest percent control was seen when a higher proportion (46%) of the *A. solani* population colonized meristems/buds of budding plants. In contrast, the lowest percent control was seen when aphids mainly colonized flowers (with only 17% of the population available on meristems). Despite being “new growth” and their greater accessibility compared to lower leaves, flowers were generally ignored by *A. aphidimyza*. These organs may be viewed as a lower-value oviposition site by this the midge, possibly due to the transient nature of flowers (pansy flowers only lasting a few days).

Reporting of percent control in our study is somewhat arbitrary, since it depends on a comparison of the treatment population to an untreated control, and is heavily affected by the intrinsic rate of increase of the aphid species in question (higher r_m_ giving a greater impression of control). Given that *M. persicae* generally has a much higher intrinsic rate of increase than *A. solani* (see Figure 4, and that the max. reported r_m_ for *A. solani* in any study at 25 °C is 0.24, while for *M. persicae* it can be upwards of 0.35 for the same temperature [31,32]), one would expect control of this species by a single release of a natural enemy to be more difficult than for a “slower growing” aphid population. That greater control of *M. persicae* was consistent in our experimentsis powerful evidence of greater attack and subsequent control of this species by *A. aphidimyza* in a multi-prey environment.

Studies with other aphid predators (e.g., [33,34]) have demonstrated that females avoid laying eggs in aphid colonies in the presence of eggs or larvae of conspecifics to mediate effects of competition and/or cannibalism. A laboratory study by Ruzicka and Havelka (1998) [35] suggested that *A. aphidimyza* also demonstrates oviposition deterrence behavior, with larval tracks limiting egg deposition. However, translation of oviposition deterrence from the lab to larger studies must be done with care. Our greenhouse experiments, where *A. aphidimyza* were allowed to oviposit over time, strongly suggest that *A. aphidimyza* females do not, in fact, adjust for conspecifics. Our results confirm those by Sentis et al. (2012) [36]. Lack of any significant oviposition deterrence surely contributes to the uneven aphid species control seen in our study, given that *A. aphidimyza* females have little reason to search for un-found aphid colonies on locations besides plant meristems.

A goal of biological control studies is often to recommend effective release rates of a predator for a specific pest. In our study, reliable control of *M. persicae* by *A. aphidimyza* was accomplished using a single release of 1 adult predator: 10 aphids. In many cases, aphid populations on individual plants were completely eliminated. With the exception of a single study on roses [8], this is the first report of release rates for this aphid predator in ornamental crops. Our levels of control were achieved using the lower-end of previously reported release rates of *A. aphidimyza* for *M. persicae* control in greenhouse vegetables, where rates have varied from a predator: prey ratio of 1:10 [25] to as high as 1:3 at 14 day intervals [37]. However, we acknowledge that in many cases the number of aphids left at the end of experiments were still unacceptable for ornamental growers, and that aphid-infested bottom leaves could act as a reservoir for re-infestation. Thus, higher rates, or multiple releases, of *A. aphidimyza* may be necessary for acceptable control. Effective release rates for *A. solani* control with *A. aphidimyza* still require much investigation. It is currently unclear if, even at higher rates or number of releases, that all *A. solani* on bottom leaves will be found by the predator. In fact, there may be some “threshold” population of *A. solani* that proves difficult to control using this particular predator, due to the aphid’s presence in cryptic, “low-quality” locations.Ultimately, *A. aphidimyza* may prove more effective against multi-species aphid infestations when prophylactic releases are made (see [38]). More testing is needed to confirm this quantitatively, though weekly or biweekly preventative releases is how this biocontrol agent is generally employed in ornamental/vegetable greenhouse crops for aphid control in North America [7]. With our current understanding of this natural enemy, under current greenhouse growing conditions, the best use of *A. aphidimyza* in a curative capacity may be to augment control by more specific aphid natural enemies (e.g., parasitoids such as *Aphidius colemani* and *A. ervi*, which are not considered 100% reliable for aphid control in greenhouse crops either) [39,40]. More research is needed into factors that optimize or reduce the performance of *A. aphidymza* in greenhouses (e.g., host-plant effects, climate, risks of intra-guild predation, etc.) before we can fully understand its most effective role within an aphid IPM program.

## 5. Conclusions

Our results illustrate the challenge in curatively controlling multi-aphid species outbreaks with a single, polyphagous aphid predator. Our study also demonstrates that prey microhabitats can play a significant role in predicting control outcomes, even with high, inundative releases of natural enemies. Although our research suggests that some control of *A. solani* is possible with *A. aphidimyza*, the presence of other aphids and crop stage have the ability to significantly affect the degree of control. We therefore suggest that careful monitoring of the less-preferred prey in a mixed-prey environment is prudent for a successful biocontrol program under these conditions. We also suggest that further tests be done in commercial operations to confirm optimal predator: prey release rates for curative control of individual, common aphid pests, given the preferences demonstrated here.

## Figures and Tables

**Table 1:** Details of experimental set-up of plant growth-stage experiments in greenhouse (GH) compartments.

Plant Stage	No. of GH Compts.	No. of Blocks (Benches) per Compt.	Treatments per Compt.	Prey spp. per Compt.	No. of Plants (Replicates) per Block per Trt./Spp. Combo.	Total No. of Plants (Replicates) per Trt./Spp. Combo.	No. of Plants Destructively Sampled per Trt./Spp. Combo. on Each Sampling Date
Vegetative	3	4	2	2	3	36	12
Budding	3	4	2	2	3	36	12
Flowering	2	4	2	2	3	24	8

**Table 2:** Statistics for aphid-infested plants treated with *A. aphidimyza* or left untreated at different stages of plant growth of pansy (*Viola × wittrockiana* Gams). Data were log(*x* + 1) transformed prior to analysis.

	Vegetative Plants	Budding Plants	Flowering Plants
Effect/Species Tested	ANOVA *F*-tests		
Model Effects			
Treatment	*F*_1,41.1_ = 68.23, *p* < 0.0001	*F*_1,41.5_ = 116.69, *p* < 0.0001	*F*_1,20.3_ = 18.86, *p* = 0.0003
Aphid Species	*F*_1,41.1_ = 0.073, *p* = 0.3965	*F*_1,41.5_ = 1.70, *p* = 0.2210	*F*_1,20.3_ = 0.14, *p* = 0.7108
Treatment × Species	*F*_1,41.1_ = 6.56, *p* = 0.0142	*F*_1,41.5_ = 21.56, *p* < 0.0001	*F*_1,20.3_ = 5.05, *p* =0.0359
